# Implementation of a multidisciplinary infections conference improves the treatment of spondylodiscitis

**DOI:** 10.1038/s41598-021-89088-5

**Published:** 2021-05-04

**Authors:** D. Ntalos, B. Schoof, D. M. Thiesen, L. Viezens, H. Kleinertz, H. Rohde, A. Both, A. Luebke, A. Strahl, M. Dreimann, M. Stangenberg

**Affiliations:** 1 Division of Spine Surgery, Department of Trauma and Orthopedic Surgery, University Medical Center Hamburg-Eppendorf, Martinistrasse 52, 20246 Hamburg, Germany; 2 Institute of Medical Microbiology, Virology and Hygiene, University Medical Center Hamburg-Eppendorf, Martinistrasse 52, 20246 Hamburg, Germany; 3 Institute of Pathology, University Medical Center Hamburg-Eppendorf, Martinistrasse 52, 20246 Hamburg, Germany; 4 Division of Orthopedics, Department of Trauma and Orthopedic Surgery, University Medical Center Hamburg-Eppendorf, Martinistrasse 52, 20246 Hamburg, Germany

**Keywords:** Clinical microbiology, Medical research, Health care, Orthopaedics, Infectious diseases

## Abstract

Establishing a multidisciplinary approach regarding the treatment of spondylodiscitis and analyzing its effect compared to a single discipline approach. 361 patients diagnosed with spondylodiscitis were included in this retrospective pre-post intervention study. The treatment strategy was either established by a single discipline approach (n = 149, year 2003–2011) or by a weekly multidisciplinary infections conference (n = 212, year 2013–2018) consisting of at least an orthopedic surgeon, medical microbiologist, infectious disease specialist and pathologist. Recorded data included the surgical and antibiotic strategy, complications leading to operative revision, recovered microorganisms, as well as the total length of hospital and intensive care unit stay. Compared to a single discipline approach, performing the multidisciplinary infections conference led to significant changes in anti-infective and surgical treatment strategies. Patients discussed in the conference showed significantly reduced days of total antibiotic treatment (66 ± 31 vs 104 ± 31, *p* < 0.001). Moreover, one stage procedures and open transpedicular screw placement were more frequently performed following multidisciplinary discussions, while there were less involved spinal segments in terms of internal fixation as well as an increased use of intervertebral cages instead of autologous bone graft (*p* < 0.001). *Staphylococcus aureus* and *Staphylococcus epidermidis* were the most frequently recovered organisms in both patient groups. No significant difference was found comparing inpatient complications between the two groups or the total in-hospital stay. Implementation of a weekly infections conference is an effective approach to introduce multidisciplinarity into spondylodiscitis management. These conferences significantly altered the treatment plan compared to a single discipline approach. Therefore, we highly recommend the implementation to optimize treatment modalities for patients.

## Introduction

Spondylodiscitis, also referred to as vertebral osteomyelitis, is a serious disease with an incidence of 2.2–5.8 per 100.000 and a mortality rate of up to 20%^1^. Main treatment goals include the elimination of the infection as well as preservation or restoration of spinal stability and neurological function^1,2^. Due to its complexity, diagnosis and treatment of spondylodiscitis remain very challenging and require a coordinated approach^2^. Surgical management consists of a wide spectrum of procedures including specimen recovery, debridement of the septic focus, instrumented stabilization, autologous bone graft or cage interposition, vertebral replacement and spinal decompression. Also, the surgical strategies in terms of approach (anterior, posterior or combined), quantity (single-stage or two-stage) and invasiveness (open and percutaneous) need to be defined^1–6^. Moreover, complicated by the emergence of highly resistant, Gram-positive and –negative organisms, duration and choice of antimicrobials may be challenging. Furthermore, conservative and additional options such as immobilization, bed rest and physical therapy have to be considered in the treatment plan^2,7,8^. Still, the optimal treatment modalities and their indications are controversial and precise recommendations are lacking^1,2,7^.

A multidisciplinary approach with involvement of infectious disease specialists to bone and joint infections is a long-standing concept. Recently it has even been stated that orthopedic infectious disease can be considered as subspecialty of its own^9,10^. Therefore clinical practice guidelines recommend a multidisciplinary approach to spondylodiscitis care that brings together all relevant disciplines to discuss optimal disease management^7,11^. However, multidisciplinary case conferences have not been introduced or examined in the context of spondylodiscitis so far while being an essential component of cancer care in many countries^11–14^. Recently, this standardized multidisciplinary approach was introduced in the context of prosthetic joint infections as well^13,15^. The implementation of these conferences could improve the treatment plan and might even be associated with better clinical outcome and survival^12–14^. In this study, we retrospectively analyzed patient records to test the hypothesis that the implementation of a multidisciplinary infections conference is an effective approach to significantly improve management of spondylodiscitis.

## Patients and Methods

### Study setting

#### Level of Evidence: III

This study was conducted at a large University Medical Center located in central Europe. The hospital is a 1600 bed tertiary care provider hosting in-house departments for medical microbiology and pathology. Patients included were treated in the spine center which is incorporated in the Department of Trauma- and Orthopedic Surgery. All methods were conducted in accordance with relevant guidelines and regulations. All experimental protocols were approved by and informed consent was obtained from all subjects according to the Ethics Committee of the University Medical Center Hamburg-Eppendorf (Hamburg Medical Chamber, Hamburg, study number WF-013/20).

#### Infections conference

In 2011 multidisciplinary infections conferences were first established at our institution and the standardized setting was as previously described^13^. Since then the conference is held on a weekly basis. Every patient diagnosed with spondylodiscitis is included in the conference and discussed every week until discharge. The conference is organized and prepared by the department of trauma and orthopedic surgery and always takes place in a same defined meeting room at the same time that allows and simplifies all specialties to participate. Four specialties need to take part in order to validate multidisciplinary decision making: a senior spinal surgeon, a senior pathologist, a senior microbiologist and an infectious disease specialist. Each case was furthermore discussed with a senior radiologist but for organizational reasons in a separate setting. In urgent medical cases the plan was discussed immediately and afterwards included in the conference and reevaluated. Apart from spondylodiscitis, prosthetic joint infections, osteomyelitis, soft tissue infections and osteosynthesis associated infections are discussed in the conference as well but with a senior orthopedic surgeon. The spinal surgeon is in charge of case selection and its presentation, orally and in written form beforehand. The presentation document is standardized and includes patient data, past medical history, risk factors, lab results, date of diagnosis, current treatment modalities, operations, clinical presentation, wound status, current antibiotics and microbiological results, if available. The duties of the pathologist include presentation of the histopathological findings, especially in terms of acute or chronic inflammatory aspects. The microbiologist is responsible for interpretation of microbiological findings, suggests performance of additional microbiological diagnostics if necessary and defines optimal antibiotic regimens. Major case discussion aspects include: type of treatment (operative vs conservative); surgical strategy; type, number and duration of antibiotics used as well as duration of the inpatient and outpatient treatment. After the discussion, the interdisciplinary treatment plan is established and delivered to each physician in charge.

### Patient selection

All patients with diagnosed spondylodiscitis (hematogenously or per continuitatem) who were treated at our institution between 2003 and 2018 were included into this retrospective analysis. Cases of postoperative spondylodiscitis were excluded. Spondylodiscitis was defined as previously described by the presence of characteristic radiological changes of the intervertebral disc and adjacent vertebrae in MRI and CT scan as well as typical clinical and laboratory findings indicating an infection (back/neck pain, fever, elevated C-reactive protein and white blood cell count). Microbiological work up included 3–5 five tissue biopsies which were collected either via open surgery or transpedicular approach (10 Gauge Jamshidi needle) as well as two set of blood cultures. Furthermore at least one sample was obtained for histopathological work up^16^. Every patient included in this study underwent spinal MRI evaluation or if MRI evaluation was not possible due to a cardiac pacemaker, CT with contrast medium or PET- CT Scan were performed instead. Overall 361 patients were identified. Patients were divided into two groups depending on if they were discussed in the infections conference (referred to as group 1; year; 2013–2018 n = 212) or not (referred to as group 2; year 2003–2011; n = 149).

### Retrospective analysis

Recorded data included age, sex, ASA score, recovered organisms, anatomic and segmental distribution of the spondylodiscitis, length of hospital and intensive care unit stay as well as therapeutic management (antibiotic and surgical strategy). Surgical data included the number of involved segments, type of screw placement (open and percutaneous), one stage or two stage procedures, as well as the type of anterior spinal fusion (intervertebral cage implantation, autologous iliac crest bone graft, vertebral body replacement, none). To analyze the antibiotic strategy, all systematically active agents were analyzed in terms of Defined Daily Dose (DDD)^17^, duration of therapy as well as mode of application (orally or intravenously).

### Statistical analysis

Mean value, range and standard deviation were calculated for all variables. Significance was tested by two tailed *t-*test and Pearson´s chi-squared test where appropriate with a p-value of < 0.05 indicating statistical significance. To determine whether significant differences are attributable to the introduction of a multidisciplinary infections conference a multivariate analysis of covariance (MANCOVA) was performed. To test for the overall difference among variables, the analysis was performed with group assignment as independent variable and time as covariate indicating a possible development trend in spinal surgery. In this mixed model approach, dichotomous variables were simultaneously included in addition to continuous variables as dependent variables. However, a classic analysis of variance could be conducted as the study includes a sufficiently large number of cases^18^. All data were analysed using SPSS software version 25.0 (SPSS, Chicago, Illinois, USA).

### Ethical approval

Ethical Review Committee Hamburg, Germany; study number: WF-013/20.

## Results

A total of 361 patients diagnosed with spondylodiscitis were included in the study. 212 patients were discussed in the infections conference (group I), whereas 149 patients were treated without conference discussion (group II). Patient characteristics are displayed in Table 1.

**Table 1:** Patient characteristics, ASA score as well as spinal localization and distribution in both treatment groups. The ASA Score in both groups differed significantly (*p* < 0.001).

	With infections conference n = 212	Without infections conference n = 149
Mean age (years)	65 ± 15 (range: 19—89 years)	65 ± 15 (range: 25—89 years)
Male to Female Ratio	2:1	1.5:1
Mean ASA Score	3.2 ± 1	2.8 ± 0,6
Median ASA Score	3 (1–4)	3 (1–5)
**Localization**		
Lumbar	113 (53%)	92 (62%)92 (62%)
Thoracic	48 (23%)	57 (38%)
Cervical	33 (16%)	0 (0%)
Multifocal	18 (8%)	0 (0%)0 (0%)
**Segmental distribution**		
Monosegmental	153 (72%)	122 (82%)
Bisegmental	32 (15%)	11%)
≥ Three segments	27 (13%)	10 (7%)

Analysis of recovered organisms revealed *S. aureus* and *S. epidermidis* to be the most common causative organisms in both groups (Table 2).

**Table 2:** Absolute and relative distribution of recovered top four organisms in both groups.

	Distribution of recovered organisms
With infections conference (n = 212)	*S. aureus* (64; 30%)
	*S. epidermidis* (39; 18%)
	*E. coli* (11; 5%)
	*M. tuberculosis* (8; 4%)
	Other (62; 29%)
	No findings (31; 15%)
Without infections conference (n = 149)	*S. epidermidis* (36; 24%)
	*S. aureus* (30; 20%)
	*E. coli* (10; 7%)
	*M. tuberculosis* (8; 5%)
	Other (76; 51%)
	No findings (17; 11%)

Focusing on a potential change in treatment plan, we analyzed the different surgical procedures performed. Whereas 70% of the patients, discussed in the infections conference, underwent a one stage strategy, only 48% were treated with this strategy when the decision was made by a single discipline approach (Table 3, *p* = 0.001). In group I, pedicle screws were mostly placed in an open manner (80%). In contrast, in over 50% of the patients of group II the screws were inserted percutaneously (Table 3, *p* < 0.001). The type and technique of spinal fusion differed significantly between the two groups (Table 3). In group I, most patients were treated with a cage interponat (44%). In contrast, only 7% of the patients in group II received a spinal cage implantation. Instead, the most common choice in group II was the implantation of an iliac crest autologous bone graft (64%) compared to 23% in group I (Table 3). Unplanned operative revision due to complications (impairment of wound healing, screw displacement, relevant postoperative hematoma, neurological deficit) was similar in both groups (group I 20%, group II 19%, *p* = 0.809).

**Table 3:** Surgical strategy and techniques in both groups.

Type of surgery	With infections conference	Without infections conference	*p*-value
**Surgical strategy**			
One stage	131/188 (70%)	67/141 (48%)	0.001
Two stage	57/188 (30%)	74/141 (52%)	0.001
**Mean number of operated segments**			
Stabilization	2.7 ± 1.8	3.7 ± 1.7	< 0.001
Decompression	1.1 ± 1.2	1.1 ± 0.9	0.84
**Transpedicular screw placement**			
Open	144/161 (89%)	73/148 (49%)	< 0.001
Percutaneous	17/ 161 (11%)	75/148 (51%)	< 0.001
**Type of anterior spinal fusion**			
Titanium or polyetheretherketone cage (PEEK)	82/188 (44%)	7/144 (5%)	< 0.001
Autologous bone graft (iliac crest)	43/188 (23%)	92/144 (64%)	< 0.001
Vertebral body replacement	24/188 (13%)	10/144 (7%)	0.083
No interponate	49/188 (26%)	35/144 (24%)	0.71

**Figure 1:**
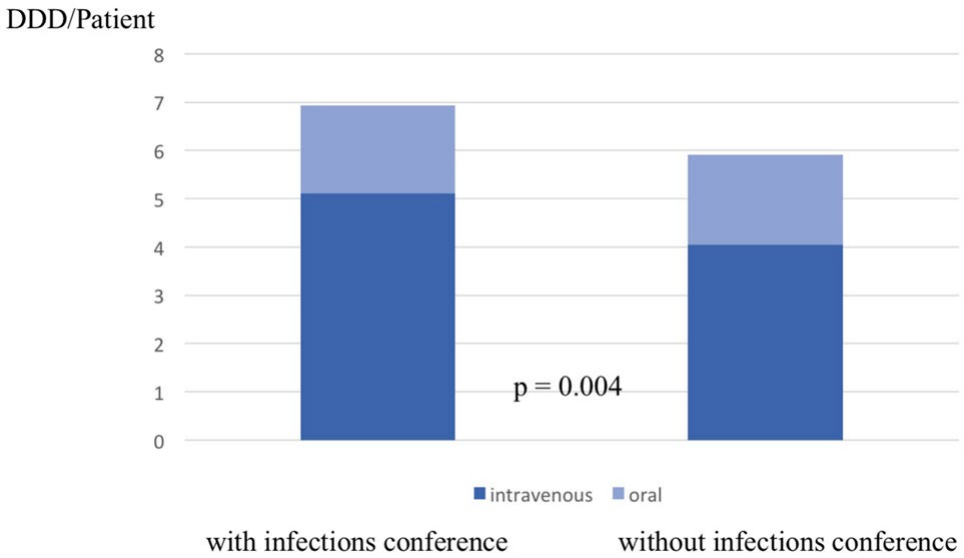
Defined Daily Dose per patient in both groups and subcategorization according to the application method. Number of patients included: 96 (without infections conference), 203 (with infections conference).

**Figure 2:**
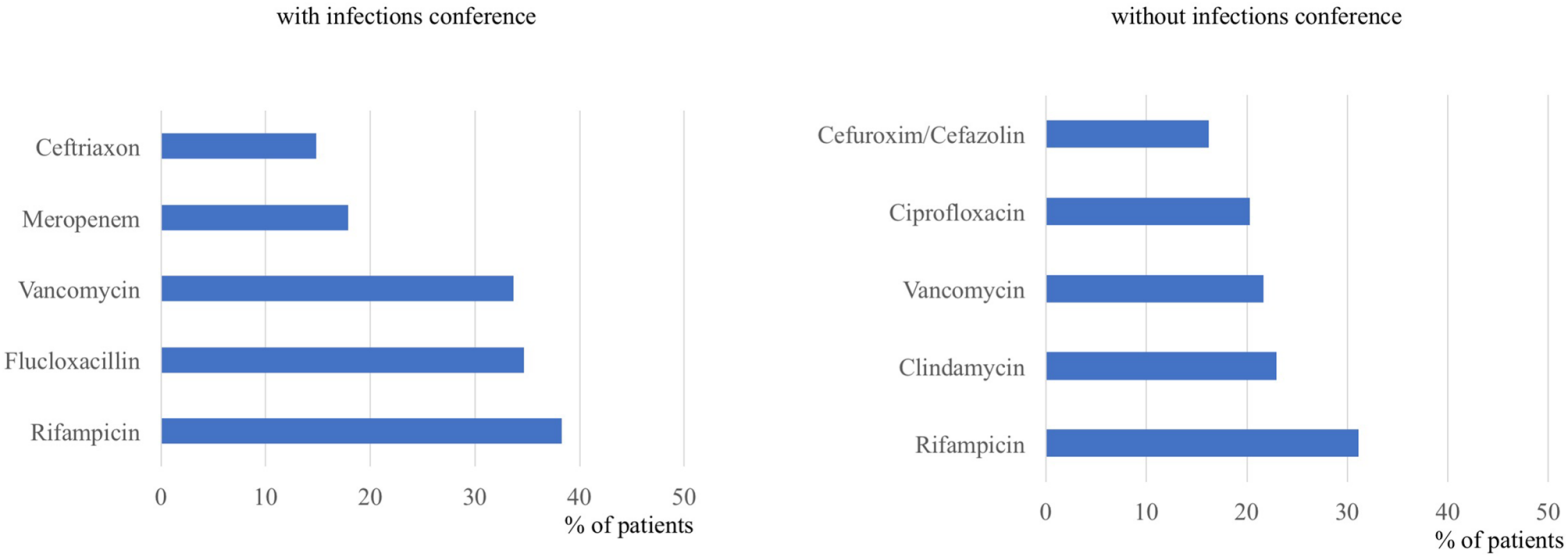
Relative distribution of top five intravenous antibiotics used in both groups. Number of patients included: 74 (without infections conference), 196 (with infections conference).

**Figure 3:**
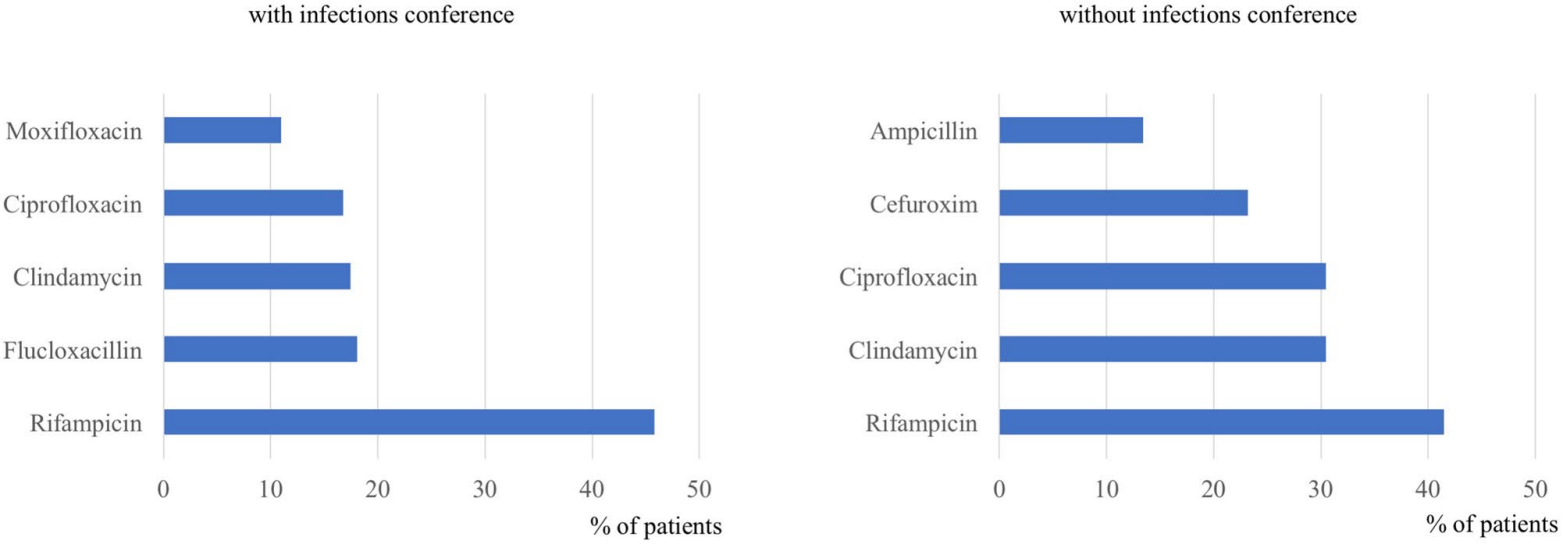
Relative distribution of top five oral antibiotics used in both groups. Number of patients included: 82 (without infections conference), 155 (with infections conference).

Overall (orally and intravenously), group I showed a significant shorter duration of antibiotic treatment (group I 66 d ± 31, group II 104 d ± 31, *p* < 0.001, Table 4). Further analysis focusing on the mode of application revealed a significantly longer intravenous therapy in group I compared to group II (Table 4). Analysis of the DDD values in both groups revealed a significant increase in the infections conference group (Fig. 1, *p* = 0.004). Regarding the type of antibiotics, Rifampicin was the most frequently used antibiotic in both groups for oral and intravenous therapy. In contrast to group II, the narrow spectrum beta-lactam antibiotic Flucloxacillin was frequently used if the decision was made by the multidisciplinary conference (Figs. 2 and 3).

**Table 4:** Mean duration in days of intravenous, oral and total antibiotic therapy. Due to its prolonged therapy, *M. tuberculosis* spondylodiscitis is not respected in this analysis.

	With infections conference	Without infections conference	*p*-value
Intravenous antibiotic therapy	26 ± 19	18 ± 8	< 0.001
Oral antibiotic therapy	40 ± 33	87 ± 25	< 0.001
Total antibiotic therapy	66 ± 31	104 ± 31	< 0.001

Comparing the total length of hospital stay between the two groups did not reveal a significant difference with a mean length of 27 days (Group I) and 31 days (Group II) retrospectively (*p* = 0.1). Still, patients who were treated according to a plan established by the infections conference (Group I) had a significantly (*p* = 0.003) longer stay on the ICU (Table 5).

**Table 5:** Mean length and standard deviation of total hospital stay in days as well as mean length and standard deviation in days of intensive care unit treatment.

	With infections conference (n = 212)	Without infections conference (n = 149)	*p*-value
Mean length of hospital stay	27 ± 21	31 ± 22	0.1
Mean length of ICU stay	7 ± 15	3 ± 9	0.003

The results of MANCOVA indicate that the main effect of the multidisciplinary infections conference was significant (F = 5.11, *p* < 0.001). The implementation had an independent significant impact on length of ICU stay (F = 4.62, *p* = 0.033), total days of oral (F = 15.99, *p* < 0.001), intravenous antibiotic therapy (F = 6.17, *p* = 0.014) and transpedicular screw placement (F = 23.06, *p* < 0.001) controlled for the variable time. There is also a trend that the surgical procedure (one-stage vs. two stage) could be influenced by the multidisciplinary conference (F = 3.67, *p* = 0.057). Differences in type of anterior spinal fusion cannot be attributed to the implemented conference. Instead, the main effect of the covariate was also significant (F = 2.31, *p* = 0.022), indicating that time exclusively had a significant effect on the change in use of a cage interponat (F = 20.33, *p* < 0.001) and iliac crest autologous bone graft (F = 4.63, *p* = 0.033) as type of anterior spinal fusion.

## Discussion

This study demonstrates the successful implementation of a weekly infections conference in the treatment of spondylodiscitis.

In the context of cancer care, multidisciplinary conferences are already a fundamental practice and these conferences can be linked to a change in treatment plan as well as improved patient outcome and survival^19–22^. Therefore, the here reported infections conference was established in a similar manner with weekly frequency, as reported in the majority of the reported studies in the literature^12,14,23,24^. Due to the attendance of at least a spinal surgeon, a microbiologist, an infectious disease specialist and a pathologist as well as the additional discussion with a radiologist, the key specialties needed in the management of spondylodiscitis are all closely involved in the treatment plan.

Similar to multidisciplinary cancer conferences our results show that performing these discussions led to a significant alteration in the treatment plan, displayed by a different surgical and antibiotic regimen. Patients in the infections conference group received a significantly shorter duration of antibiotic treatment, which was furthermore strengthened by a multivariate analysis emphasizing the impact of the conferences. The results are in accordance to the recent opinion that a shorter period of antibiotic treatment (mostly 6 weeks) is not inferior to a longer application duration (mostly 12 weeks) and reflects the current guidelines of the Infections Disease Society of America^11,25–27^. Establishing these conferences could therefore be a useful instrument to secure adherence to guidelines, as numerous studies have demonstrated improved guideline adherence due to the implementation of multidisciplinary cancer conferences^22,23,28,29^. Limiting the duration of antibiotic therapy to a shorter but equally effective course may further the goal of reducing side effects in the individual patient and reducing overall antibiotic consumption, thus reducing the development and selection of drug resistant bacteria^30–32^. Interestingly, the intravenous treatment duration was significantly increased in the infections conference group, which could be explained by the prolonged intensive care unit stay and higher ASA Scores of patients in this group. However, compared to most other studies the duration of intravenous therapy in both groups of this study is comparatively low^11,33–35^. Furthermore, potentially improved bioavailability might influence the slight increase of duration as well, especially when taking the limited bone penetration of most antibiotics and the prolonged bone revascularization after surgery into consideration. Moreover, the increased rate of *S. aureus* in the conference group could be another explanation since *S. aureus* infections are thought to be associated with higher complications so that prolonged intravenous therapy could be essential^1,36–39^. Still, it needs to be considered that there is a lack of data supporting its efficacy and recent research questions the superiority of intravenous compared to oral antibiotic treatment but not in the specific context of spondylodiscitis^11,40–42^. Even though several surgical and instrumentation techniques to treat spondylodiscitis have been described, valid recommendations are few and there is no consensus about the optimal surgical strategy. The multidisciplinary conference is a tool to strengthen a standardized approach while also respecting the heterogeneous and individual nature of this disease. Even though we could detect significant alterations regarding the surgical management, multivariate analysis revealed that only certain aspects can be attributed to the conference. It has to be considered, that surgical strategies as well as implants have changed over the years, so that it is to be expected that the recorded differences cannot just be ascribed to the conference discussion. In this study, the alterations in the surgical strategy which can be associated to the conference by multivariate analysis were the type of screw placement as well as an almost significant trend towards one-stage procedures. Whereas the type of anterior spinal fusion could not be attributed to the implemented conference.

A potential change in treatment plan due to these conferences is one of the most valuable arguments in favor of conducting these conferences which is supported by these results^14,23^. Still, even though therapeutic changes could be demonstrated in this study we could not detect any changes regarding complications leading to operative revision or when it comes to the total length of hospital stay, as previously reported in other studies^13,14^. Therefore, a change in clinical outcome could not be shown in this study.

Additionally, following implementation of the infections conferences, it was noticeable that they were an efficient tool to accelerate the process of multimodal decision making, which both the patient and the staff benefitted from. Especially complex medical decisions which rely on fundamental knowledge in various fields can be quite time consuming for hospital staff. Consulting physicians might not be readily available and it can be an administrative challenge to gather all information and implement the expert opinions of various people. With all fields of expertise gathered at the weekly conferences, complex decisions could be efficiently and profoundly made.

The limitations of the study include its retrospective nature and a lack of randomization. Also, the different distribution of spinal localization and different ASA scores in the two groups lead to a potential bias. It needs to be recognized that, according to multivariate analysis, not all significant results could be attributed to the introduction of the conference. The results of our study do not allow to comment on prognostic outcome and need to be carefully interpreted.

In conclusion, due to the successful implementation of a multidisciplinary infections conference our study demonstrates a feasible and effective solution to ensure the multidisciplinary approach in spondylodiscitis care. Performing these conferences significantly alters the treatment plan when compared to a single discipline approach and is a helpful tool to increase the efficiency of multidisciplinary decision making. Prospective studies are warranted in order to examine the conference´s effect on clinical outcome.

## Data Availability

All data generated or analyzed during this study are included in this published article.
